# Multiplex-Ready PCR: A new method for multiplexed SSR and SNP genotyping

**DOI:** 10.1186/1471-2164-9-80

**Published:** 2008-02-18

**Authors:** Matthew J Hayden, Thao M Nguyen, Amanda Waterman, Kenneth J Chalmers

**Affiliations:** 1 Molecular Plant Breeding CRC, PMB 1, Glen Osmond, SA, 5064, Australia; 2 School of Agriculture, Food and Wine, Adelaide University, Waite Campus, Urrbrae, 5064, Australia

## Abstract

**Background:**

Microsatellite (SSR) and single nucleotide polymorphism (SNP) markers are widely used in plant breeding and genomic research. Thus, methods to improve the speed and efficiency of SSR and SNP genotyping are highly desirable. Here we describe a new method for multiplex PCR that facilitates fluorescence-based SSR genotyping and the multiplexed preparation of DNA templates for SNP assays.

**Results:**

We show that multiplex-ready PCR can achieve a high (92%) success rate for the amplification of published sequences under standardised reaction conditions, with a PCR specificity comparable to that of conventional PCR methods. We also demonstrate that multiplex-ready PCR supports an improved level of multiplexing in plant genomes of varying size and ploidy, without the need to carefully optimize assay conditions. Several advantages of multiplex-ready PCR for SSR and SNP genotyping are demonstrated and discussed. These include the uniform amplification of target sequences within multiplexed reactions and between independent assays, and the ability to label amplicons during PCR with specialised moieties such fluorescent dyes and biotin.

**Conclusion:**

Multiplex-ready PCR provides several technological advantages that can facilitate fluorescence-based SSR genotyping and the multiplexed preparation of DNA templates for SNP assays. These advantages can be captured at several points in the genotyping process, and offer considerable cost and labour savings. Multiplex-ready PCR is broadly applicable to plant genomics and marker assisted breeding, and should be transferable to any animal or plant species.

## Background

Molecular markers based on the polymerase chain reaction (PCR) are widely used in plant breeding and genetic research. For example, they are the basis for the mapping of genes and quantitative trait loci (QTL), marker assisted breeding, phylogenetic studies and comparative genomics [1-4]. In particular, two types of marker are best suited for these applications: single nucleotide polymorphisms (SNPs) and microsatellites (SSRs). To date, almost all methods published for SNP detection in plants consist of two steps: amplification (usually by PCR) of a target sequence harbouring the polymorphism, followed by detection of the SNP. Methods based on hybridization, polymerization, ligation and nucleolysis have been used to interrogate the SNP (see [5-7] for reviews). In contrast, capillary and gel electrophoresis coupled with fluorescence-based detection is the most commonly reported method for the assay of SSRs.

Methods to improve the speed and efficiency of SNP and SSR genotyping are integral to the application of molecular markers in plant breeding and research. Multiplex PCR, in which several markers are simultaneously amplified in the same reaction, is used to increase the amount of information generated per assay, and to reduce consumable and labour costs [8]. The M13-tailed primer method [9] is often used for the assay of SSRs to reduce the cost of fluorescent primer labelling, which is typically five to ten times more expensive than the synthesis of an unlabeled primer. In this three primer strategy, PCR is performed using a forward primer with a nucleotide extension at its 5'-end, identical to the sequence of an M13 sequencing primer, a standard length reverse primer and a fluorescently labelled M13 primer. During PCR, the SSR product is fluorescently labelled following participation of the M13 primer after the first few cycles of amplification. Therefore, instead of synthesizing one specific fluorescently labelled primer for each SSR marker, only a dye labelled M13 primer is needed. The M13-tailed primer method has also been used to modify target sequences harbouring SNPs during PCR amplification [10,11].

The development of multiplex PCR assays in plants can be difficult due to large genome sizes and polyploidy. For example, extensive optimisation is typically required for multiplex SSR amplification in bread wheat [12], one of the world's most important cereal crops. Bread wheat is an allohexaploid with a large and complex genome, comprised of paralogous gene families and about 75% repetitive DNA [13]. The routine development of multiplex PCR assays in crop species with smaller diploid genomes can also be challenging [14,15]. The bottlenecks for multiplex PCR are mainly undesirable primer-primer interactions and non-specific amplification [16]. Similarly, the use of a tailed forward primer and a standard length reverse primer in the M13-tailed primer method can promote the amplification of non-specific products. Therefore, the PCR conditions required for amplification using the M13-tailed primer method are often different to those optimal for amplification using standard length primers.

Here, we present a new method for multiplex PCR that simplifies assay development and provides several technological advantages that facilitate fluorescence-based SSR genotyping and the multiplexed preparation of DNA templates for SNP detection. This method, termed multiplex-ready PCR, combines the advantages of the M13-tailed primer method and multiplex PCR in a single step, closed tube assay. It does not rely on expensive reagents, is suitable for the amplification of any published sequence and requires only the synthesis of primers in the multiplex-ready format for its deployment. Multiplex-ready PCR is evaluated in three plant genera with different genome sizes and ploidy: *Prunus *spp. (apricot and cherry), *Hordeum *spp. (barley) and *Triticum *spp. (bread wheat), which have genome sizes of about 300, 5200 and 16000 mega base pairs (Mbp), respectively.

## Results

### Evaluation of multiplex-ready PCR

**Figure 1:**
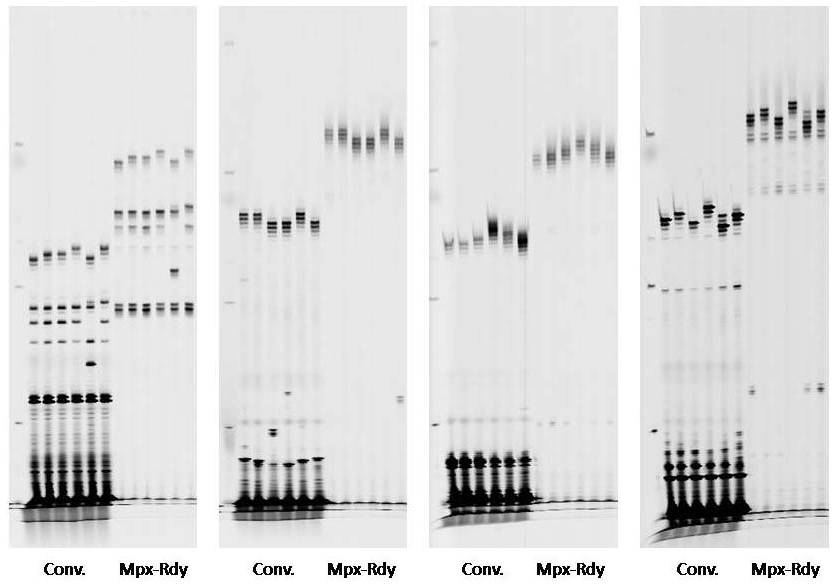
**Amplification of SSRs from six bread wheat varieties in uniplex reactions using conventional and multiplex-ready PCR**. Markers from left to right are gwm301, gwm340, gwm389 and gwm513. Multiplex-ready PCR assays were performed using 80, 70, 40 and 40 nM of locus-specific primer, respectively. Conventional PCR was performed with T_a _55, 60, 60 and 60°C, respectively. PCR products were separated on a GelScan2000 instrument.

To assess the amplification of target sequences using multiplex-ready PCR, published SSR primer sets for apricot, cherry, barley and bread wheat were tested. Initially, we determined the optimal concentration of locus-specific primer that was required to achieve strong amplification of a PCR fragment(s) of the expected size. Adjustment of the locus-specific primer concentration for individual primer sets was found to be necessary for satisfactory amplification yield and to reduce non-specific amplification. A subset (64) of the published SSRs were also amplified in uniplex reactions by conventional and multiplex-ready PCR, where conventional PCR was performed using optimal conditions for each marker. These results showed that the PCR specificity and genotyping accuracy of both methods was indistinguishable, except for a 30-bp fragment size offset resulting from the addition of the tag primer sequences to the 5'-ends of the multiplex-ready PCR products (Figure 1).

To date, we have achieved a 92% success rate for the multiplex-ready amplification of more than 2800 published SSRs under standardised reaction conditions that differ only by the concentration of locus-specific primer [17]. A similar success rate has also been achieved for the amplification of barley sequences harbouring published SNPs. The SSRs and SNPs used in the present study are described in Additional File 1.

### Development of multiplexed assays

Uniform product abundance is an important feature of any multiplex amplification method, especially for highly paralleled SSR genotyping and the preparation of samples for SNP detection assays. To determine if multiplex-ready PCR amplifies a similar amount of product for each primer set within a multiplexed assay, and between independent reactions, two experiments were performed.

In the first experiment, we performed 32 six-plex PCR assays in each of apricot and cherry, and 32 four-plex and 48 six-plex PCRs in each of barley and bread wheat using published SSR primer sets and six to eight genetically diverse lines. The choice of four- and six-plex assays was due to the limited size range of PCR fragments amplified by the published SSRs and the requirement to construct marker panels with non-overlapping allele sizes. The amount of SSR product amplified in the multiplexed reactions was assessed following ABI3730 separation using GeneMapper software by measuring the fluorescence intensity for each marker. A standardised procedure was used to prepare the SSR products for electrophoresis to ensure that the fluorescence intensity observed for each SSR was representative of its amplification yield within a multiplexed reaction, and to allow marker yield between independent assays to be compared (see *Materials and Methods*).

**Figure 2:**
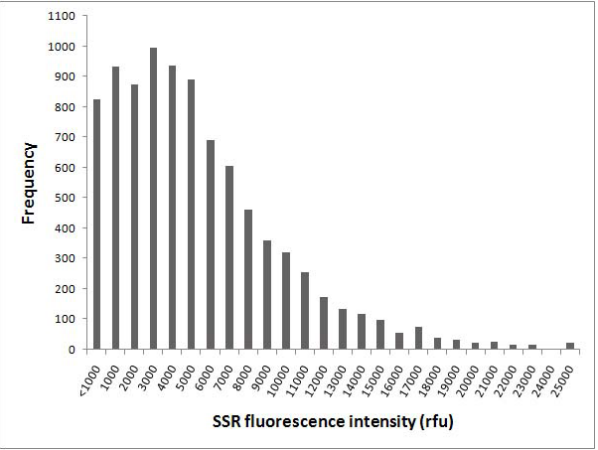
**Distribution of ABI3730 fluorescence intensities for published SSRs amplified in four- and six-plex multiplex-ready PCR assays**. A total of 8960 SSR loci were amplified in 32 six-plex PCRs from each of six apricot and cherry varieties, and 32 four-plex and 48 six-plex PCRs from each of eight barley and bread wheat varieties.

Frequency distribution analysis showed that 85% (1045/1216) of the SSRs present in the multiplexed PCRs were successfully amplified, as assessed by ABI3730 fluorescence intensities that fell within a range optimal for semi-automated allele sizing, i.e. 1000–15000 relative fluorescence units (rfu). Sixty eight percent of the SSR alleles had a fluorescence intensity within the range of 2000–10000 rfu. These results indicated that a scorable amount of product was amplified for each primer set within the multiplex PCRs and between independent reactions (Figure 2). Visual inspection of the ABI electrotraces revealed that the main cause of variation in fluorescence intensities was SSR stutter bands, with markers producing stutter bands typically having lower fluorescence intensity than those with no or few stutter bands.

**Figure 3:**
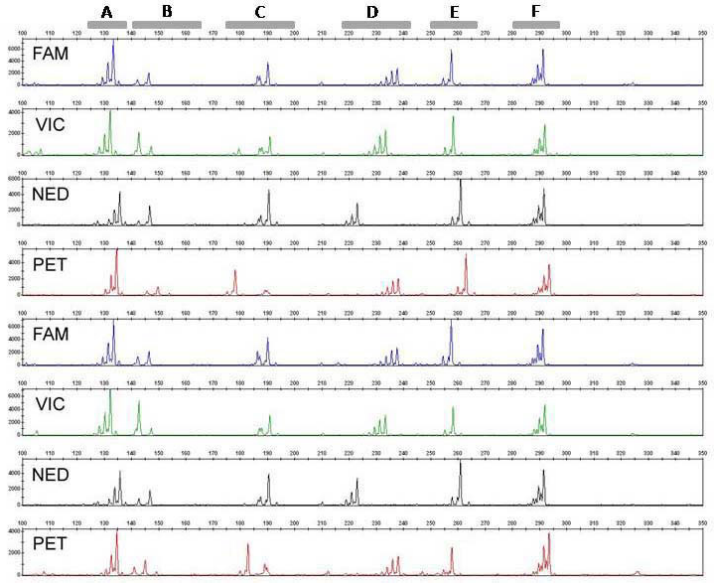
**ABI3730 electrotraces showing six-plex PCRs amplified from eight bread wheat varieties**. Multiplex-ready PCR was performed using the SSR markers (A) barc216, (B) barc64, (C) barc108, (D) gwm681, (E) barc273 and (F) cfa2028, and 100, 70, 120, 70, 120 and 30 nM of locus-specific primer, respectively.

**Figure 4:**
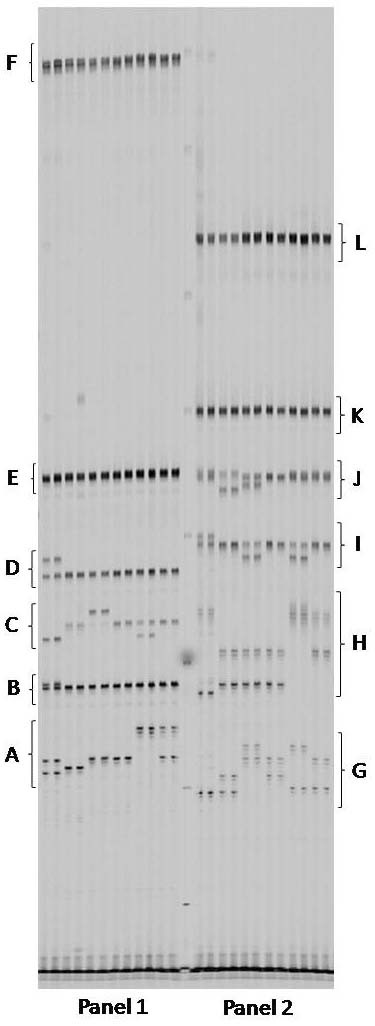
**Six-plex PCRs performed in duplicate for six apricot varieties**. Multiplex-ready PCR for panel 1 was performed using SSR markers (A) UDP96-003, (B) UDP96-008, (C) BPPCT028, (D) BPPCT014, (E) pchgms21.2 and (F) pchgms17 with 50, 50, 40, 40, 70 and 50 nM of locus-specific primer, respectively; and for panel 2 using SSR markers (G) UDP96-010, (H) BPPCT039, (I) BPPCT004, (J) pchcms04, (K) pchgms11.2 and (L) pchgms23 with 60, 50, 50, 70, 70 and 70 nM of locus-specific primer, respectively. The SSR products were separated on a Gel Scan 2000 instrument.

Of the 64 six-plex PCRs performed in apricot and cherry, 36 (56%) amplified all six SSRs and 26 (41%) amplified four or more SSRs (Table 1). Of the 48 six-plex PCRs performed in each of barley and bread wheat, 19 (40%) and 13 (27%) amplified all six SSRs, and 23 (48%) and 26 (54%) amplified four or more SSRs, respectively. An improved success rate was observed for the four-plex PCRs tested, with 24 (75%) and 22 (69%) amplifying all four SSRs in barley and bread wheat, respectively. SSRs were considered to have failed when they did not produce fluorescence intensities within the optimal range for all of the DNA samples tested, or failed to amplify PCR fragments of the allele sizes observed in uniplex reactions for the same DNA samples. Non-specific fragments were observed in some cases but these did not interfere with marker scoring. In general, the PCR specificity achieved in the multiplex PCRs was sufficient to allow unambiguous SSR scoring on a number of detection platforms (Figures 3 and 4).

**Table 1:** Success rate for the amplification of target sequences in multiplex-ready and conventional multiplex PCR assays

			Number of primer sets successfully amplified						
			
		# assays	6	5	4	3	2	1	0
***Multiplexed SSR Assays***									
6-plex PCRs	Apricot	32	17 (53%)	10 (31%)	3 (10%)	1 (3%)	1 (3%)	.	.
	Cherry	32	19 (59%)	9 (28%)	4 (13%)	.	.	.	.
	Barley	48	19 (40%)	19 (40%)	4 (8%)	4 (8%)	2 (4%)	.	.
	Bread wheat	48	13 (27%)	18 (38%)	8 (17%)	7 (14%)	1 (2%)	1 (2%)	.
									
4-plex PCRs	Barley	32	.	.	24 (75%)	6 (19%)	2 (6%)	.	.
	Bread wheat	32	.	.	22 (69%)	10 (31%)	.	.	.
									
***Multiplexed Barley SNP Assays***									
6-plex PCRs	Multiplex-Ready	24	6 (25%)	10 (42%)	5 (21%)	3 (12%)	.	.	.
	Conventional	24	.	.	3 (12%)	5 (21%)	11 (46%)	3 (12%)	2 (8%)
									
4-plex PCRs	Multiplex-Ready	24	.	.	19 (79%)	5 (21%)	.	.	.
	Conventional	24	.	.	3 (12%)	9 (38%)	7 (29%)	5 (21%)	.

In the second experiment, we performed a total of 48 four- and six-plex PCRs to amplify DNA sequences harbouring published SNPs from 16 barley lines with known SNP genotypes determined from uniplex reactions. To allow the success rate for the amplification of target sequences in multiplex-ready PCR to be quantified, the assays were also performed using conventional multiplex PCR and standard length primers. Both types of multiplex PCR assay were performed under standardised conditions (see *Materials and Methods*). The success of each multiplexed reaction was determined by assaying the SNPs harboured in the resulting PCR products and assessing the concordance of the SNP genotypes with those determined in uniplex reactions.

Overall, 95% (91/96) and 80% (115/144) of the SNP loci assayed in the reaction products of the four- and six-plex multiplex-ready PCRs were successfully detected, respectively, with complete genotype concordance between uniplex and multiplex reactions. Sixteen (67%) of the 24 six-plex PCRs amplified five or more SNP loci, and all of the 24 four-plexes amplified three or more SNP loci. In contrast, only 60% (58/96) and 36% (52/144) of the SNP loci assayed in the reaction products of the four- and six-plexes performed using conventional multiplex PCR were successfully detected, respectively. None of the 24 six-plex PCRs amplified five or more SNP loci, and only half of the four-plex assays amplified three or more SNP loci (Table 1). This corresponded to an average success rate of 88% for the amplification of target DNA sequences in the multiplex-ready PCR assays, compared to 48% for the conventional multiplex PCR assays.

## Discussion

While the use of molecular markers in plant breeding and research is well documented [18,19], the application of multiplex PCR in plants is not widely reported due to the difficulties associated with assay development. New approaches to simplify the development of multiplex PCR assays to improve genotyping throughput and reduce costs for SNP and SSR analysis have the potential to increase the speed and efficiency of plant breeding and genetic research.

### Principles of multiplex-ready PCR

**Figure 5:**
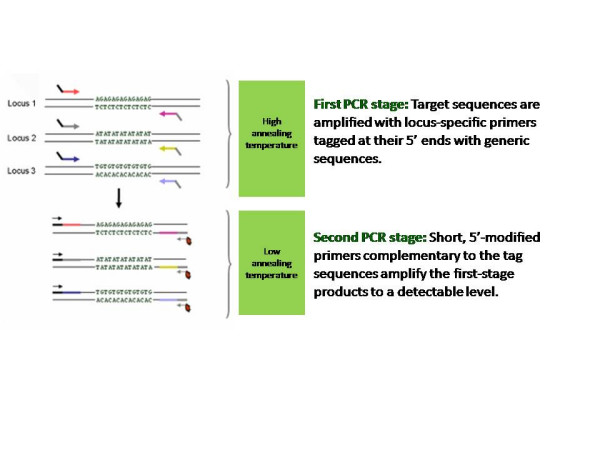
Diagrammatic representation of multiplex-ready PCR.

Multiplex-ready PCR combines the principles of the M13-tailed primer method [10] and two-step multiplex PCR amplification [11,20]. The assay comprises a single-step, closed-tube reaction in which PCR amplification takes place in two stages (Figure 5).

In the first stage, low concentrations of locus-specific primers tagged at their 5'-ends with generic nucleotide sequences amplify the target loci from genomic DNA. The locus-specific primers become fully incorporated into PCR product, which have the generic nucleotide tag sequences at their 3'- and 5'-ends. These nucleotide sequences serve as primer binding sites for the second stage of PCR, and help to reduce amplification bias between amplicons by normalizing primer hybridization kinetics [11,21]. This provides for more uniform amplification of the target sequences during the second stage of amplification, and results in a similar yield of PCR product for each target locus within a multiplexed reaction.

In the second stage of amplification, universal primers corresponding to the nucleotide tag sequences amplify the first stage PCR products to a detectable level. Restricting the participation of the tag primers to the second stage of PCR is a large difference (ca 10°C) in the annealing temperatures of the tag and locus-specific primers. The second stage amplification products can be labelled at one or both ends with specialised moieties, such as fluorescent dyes and biotin, by use of 5'-modified tag primers. Use of an excess of PCR cycles (exhaustive conditions) ensures that the tag primers become fully incorporated into PCR product to generate a similar yield of amplicons between independent assays.

### Amplification under standardised reaction conditions

A practical advantage of multiplex-ready PCR is the ability to perform assays under standardised conditions. Conventional multiplex PCR typically requires that primer sets are grouped according to their optimal annealing temperature, and often other factors such as Mg^2+ ^requirements. Published SSRs for many plant species have a wide range of optimal annealing temperatures ranging from 45 to 60°C, which results primarily from the limited availability of DNA sequence from which primer sets can be developed [22]. Differences in PCR requirements can limit the potential to perform conventional multiplex PCR for a given set of markers.

The ability to perform multiplex-ready PCR under standardised reaction conditions enables any combination of markers to be deployed for multiplexed amplification, and can improve assay throughput by facilitating PCR automation. Standardised reaction conditions are achieved by performing an initial optimisation step for each primer set, in which the optimal concentration of locus-specific primer for the amplification of the target sequence is determined. Adjusting the locus-specific primer concentration enables the PCR specificity and yield of individual primer sets to be controlled, as it overcomes differences in the melting temperature and efficiency of polymerization of each primer set on genomic template. These conditions also help to prevent undesirable amplification resulting from non-specific annealing of the locus-specific primers during the first few PCR cycles. While this initial optimisation step is time-consuming, in our experience, this process is essentially the same as that typically required for any new primer set to be used in conventional PCR. Moreover, once determined, the optimal locus-specific primer concentration is used in multiplex-ready PCR, irrespective of whether uniplex or multiplex assays are performed. This assay feature distinguishes multiplex-ready PCR from conventional multiplex PCR, where the primer concentration for each target sequence is specifically optimised for each multiplex assay.

### Development of multiplex PCR assays

The routine development of multiplex PCR assays in both animals and plants becomes more difficult as the level of multiplexing increases due to the increased chance of undesirable primer-primer interactions [16]. In plants, this is compounded by large genome sizes, polyploidy and limited marker choice. Higher multiplexing levels are generally only achieved with careful assay optimisation [23], reliance on commercially available and expensive PCR reagents [24,25], or the use of a preamplification step to first enrich the target sequences [26,27].

In the present study, a moderate success rate was achieved for the routine development of multiplexed assays in three plant genera with different genome sizes and ploidy. This success rate is measurable in two ways. First, five or more SSRs were amplified in 84, 87, 80 and 65% of the six-plex PCRs tested in apricot, cherry, barley and bread wheat, respectively. This success rate was achieved using random sets of markers chosen only for six-plex PCR because of non-overlapping allele sizes. In contrast, the achievement of a similar level of multiplexing in plants using conventional multiplex PCR is typically reported to require extensive assay optimisation, arising from the requirement to adjust the primer concentration for individual markers in each reaction [14,15]. This optimisation process is highly empirical and time consuming, and depending on the combinations of markers may or may not lead to successful assay development. And second, multiplex-ready PCR was almost two times more successful (88% versus 48%) at amplifying sequence-tagged-sites harbouring SNPs from barley in four-plex and six-plex assays, compared to conventional multiplex PCR. As expected from published literature, the multiplexing success of multiplex-ready PCR diminished with increasing genome size and polyploidy, with the success rate highest for apricot and cherry, followed by barley then bread wheat (Table 1). Apricot and cherry have small diploid genomes (ca. 300 Mbp) about twice the size of the *Arabidopsis *genome [28,29], barley has a large diploid genome of about 5200 Mbp, and bread wheat has an allohexaploid genome of around 16,000 Mbp [30].

We believe that the improved amenability of multiplex-ready PCR for multiplexed amplification results from the use of low concentrations of locus-specific primer, which helps to reduce undesirable primer-primer interactions, and an excess of PCR cycles to ensure that the locus-specific primer is completely incorporated into PCR product at the end of the first stage of amplification. The latter helps to ensure that a similar amount of PCR product is amplified for each locus before the second stage of amplification. Similarly, the amplification of a relatively constant amount of PCR product between independent reactions results from the use of an excess of PCR cycles in the second stage of amplification to ensure that the tag primer is fully incorporated into PCR product.

### Advantages of multiplex-ready PCR for SSR genotyping

Fluorescence-based SSR detection and allele sizing on an automated DNA fragment analyser is one of the fastest and most accurate methods for SSR genotyping [31,32]. This procedure is based on the separation of fluorescently labelled SSR amplicons by capillary or gel electrophoresis. An advantage of fluorescence-based SSR genotyping is that several SSRs can be simultaneously separated in a single capillary or gel lane providing that the SSR fragments have non-overlapping allele sizes. In instances where SSR allele sizes are overlapping, coseparation can be achieved by labelling the SSR products with fluorescent dyes that have different emission wavelengths. These advantages facilitate genotyping throughput by providing compatibility with multiplex PCR and multi-pooling strategies, the latter of which involves the pooling of individual PCR assays for two or more SSRs prior to electrophoresis [33].

Highly paralleled SSR genotyping on an automated DNA fragment analyser relies on the fluorescence intensities of markers falling within a range that is optimal for accurate allele detection and automated allele sizing. Ideally, the fluorescence intensity for each SSR should be relatively uniform. This is especially important for the coseparation of SSR markers with overlapping fragment sizes that are labelled with different fluorophores. Large differences in SSR fluorescence intensities can cause genotyping inaccuracies due to the inability of genotyping software to fully correct for background fluorescence, which results from the overlap of the emission spectra of different fluorophores. This phenomenon, known as "spectral bleed" and "pull-up", results in the detection of a SSR allele where no dye-labelled amplicon actually exists.

Multiplex-ready PCR provides several technological advantages for highly paralleled, fluorescence-based SSR genotyping. First, relatively uniform SSR amplification within a multiplexed assay and between independent reactions (Figure 2) enables the use of a standardised protocol to prepare SSR products for electrophoresis without the risk of under- or over-loading the amount of PCR product for individual markers. It also facilitates semi-automated allele scoring, since problems associated with "pull up" are reduced. Secondly, the ability to dye label SSR amplicons with a fluorophore of choice during PCR amplification (see Figure 3) provides flexibility for the separation of markers, since SSRs which cannot be multiplexed due to overlapping allele sizes can be coseparated by labelling with different fluorophores and multi-pooling. These two advantages can improve genotyping throughput and facilitate assay automation. A third advantage is the substantial cost savings for fluorescent primer labelling, since the synthesis of a specific fluorescently labelled primer for each SSR marker is not required. Rather, multiplex-ready PCR requires only four dye labelled primers to fully utilise the genotyping capabilities of an ABI3730 DNA fragment analyser. In this sense, multiplex-ready PCR combines both the advantages of the M13-tailed primer method and multiplex PCR for fluorescence-based SSR genotyping. The optimisation of more than 2800 barley and bread wheat SSRs for multiplex-ready PCR, and its application for highly paralleled, fluorescence-based SSR genotyping is described elsewhere [17,34].

### Advantages of multiplex-ready PCR for SNP assays

A large number of assay chemistries and analysis platforms have been described for SNP genotyping [reviewed by [5-7]]. In plants, the assay of SNPs usually consists of two-steps: PCR amplification of a target sequence harbouring the polymorphism, followed by detection of the SNP. Preamplication of the target sequence is generally required due to the large size of plant genomes and polyploidy, which can cause poor sensitivity and specificity when SNPs are assayed directly from genomic template [7]. Preamplification adds significant cost to the assay of SNPs, often requires a purification step to remove unincorporated dNTP and primer, and can be a bottleneck for high throughput genotyping when several SNPs distributed throughout the genome are assayed per sample.

Multiplex-ready PCR provides several advantages for the multiplexed preparation of DNA templates for SNP detection and offers the potential to increase assay throughput and reduce labour and consumable costs. The amplification of a similar amount of PCR product under standardised conditions for each target sequence within a multiplexed assay, and between independent reactions, provides similar advantages for assay automation to those that can be captured for SSR genotyping. Similarly, the ability to label the target sequences with specialised moieties such as biotin during PCR amplification facilitates low cost compatibility with automated procedures that can be used to purify and prepare the DNA templates for SNP detection. Multiplex-ready PCR could also potentially be modified to provide compatibility with particular types of SNP assay chemistries. For example, a T7 RNA transcription site could be introduced into the nucleotide sequence of one of the tag primers to allow the synthesis of RNA from multiplex-ready PCR products using T7 RNA polymerase to serve as template in the reverse transcription allele-specific primer extension assay described by Pastinen et al. [35].

## Conclusion

Multiplex-ready PCR is a method that simplifies the development of multiplexed PCR assays, and provides several potential advantages for fluorescence-based SSR genotyping and the multiplexed preparation of DNA templates for SNP detection assays. It differs from other published methods for multiplex PCR in two ways. First, target DNA sequences are amplified in two distinct stages that are separated by primer annealing temperature in a single-step, closed-tube reaction. And second, assay development requires only a single optimisation step to determine the optimal concentration of locus-specific primer that is used in all assays, irrespective of the level of multiplexing. Multiplex-ready PCR is broadly applicable to plant genomic research and marker assisted breeding, and should be transferable to any animal or plant species.

## Methods

### Plant Materials

Genomic DNA was extracted from frozen leaf material of apricot (*Prunus armeniaca*) and cherry (*P. persica*) using a modified CTAB method [30], and from freeze-dried leaf material of barley (*Hordeum vulgare*) and bread wheat (*Triticum aestivum*) as described by [36]. The apricot lines used were Hasanbey, Trevatt, Rivergem, Patterson, 16822 and 6431. The cherry lines were Sir Douglas, Dame Roma, Sir Tom, Sir Hans, Dame Nancy and Sir Don. The barley lines were Alexis, Chebec, Clipper, Flagship, Harrington, Haruna Nijo, Sahara3771 and Sloop. The bread wheat lines were Barunga, VPM Cook, Chinese Spring, Gabo, Norin10, Olympic, Opata85 and WI7984 (a synthetic hexaploid wheat).

### Primer synthesis

Published primer sets for SSRs and sequence-tagged-sites (STSs) harbouring SNPs were synthesised with generic non-complementary nucleotide sequences at their 5'-ends. Specifically, the forward and reverse primer for each marker was synthesised with the nucleotide sequence 5' ACGACGTTGTAAAA 3' and 5' CATTAAGTTCCCATTA 3', respectively. Primer aliquots for each marker were prepared by mixing equimolar amounts of appropriate forward and reverse primers in 1× TE (1 mM EDTA, 10 mM Tris-HCl, pH 8.0), and are hereafter referred to as locus-specific primers. Two generic tag primers, *tagF *and *tagR*, with the sequences 5' ACGACGTTGTAAAA 3' and 5' CATTAAGTTCCCATTA 3', respectively, were also synthesised. The *tagF *primer was labelled at its 5'-end with one of the following fluorescent dyes: VIC, FAM, NED and PET (Applied Biosystems).

### Primer optimisation

The optimal concentration of locus-specific primer required to amplify the target sequence was determined empirically. Initially 20, 30, 40 and 80 nM of locus-specific primer was tested. PCR products were separated on a GelScan2000 instrument (see *SSR analysis *section) or on 2% agarose gels stained with ethidium bromide. The optimal primer concentration was determined by visual inspection as the strong amplification of a PCR fragment of the expected size. In instances where it was desirable to improve PCR specificity and yield, additional locus-specific primer concentrations were tested.

### Multiplex-ready PCR assays

The amplification of published SSRs and sequences harbouring SNPs by uniplex and multiplex PCR was performed under identical reaction conditions. PCR was performed in a 6 μl reaction mixture containing 0.2 mM dNTP, 1× ImmoBuffer (Bioline) (16 mM (NH_4_)_2_SO_2_, 0.01% Tween-20, 100 mM Tris-HCl, pH 8.3), 1.5 mM MgCl_2_, 100 ng/μl bovine serum albumin Fraction V (Sigma Aldrich), 75 nmol each of dye-labelled *tagF *and unlabeled *tagR *primer, 50 ng genomic DNA, 0.15 U Immolase DNA polymerase (Bioline), and an appropriate concentration of locus-specific primer (see Additional File 1). For multiplex PCR, locus-specific primers for several markers were added to each reaction at the optimal concentration determined in uniplex assays. Due to limited marker choice, some redundant use of primer sets amplifying SSRs and sequences harbouring SNPs was required to construct the multiplex panels. Following an initial denaturation step of 10 min at 95°C to heat activate the DNA polymerase, PCR was performed for a total of 65 cycles with the profile: 30 s at 92°C, 90 s at 50°C, 60 s at 72°C for five cycles. The next 20 cycles were 30 s at 92°C, 90 s at 63°C, and 60 s at 72°C, followed by 40 cycles with 15 s at 92°C, 30 s at 54°C, and 60 s at 72°C, and a final extension step of 10 min at 72°C. The purpose of the initial five cycles with 50°C annealing was to improve the amplification efficiency of published primer sets with low (< 50°C) annealing temperature. Preliminary studies showed that the amplification yield of such primers could be improved without significantly increasing the locus-specific primer concentration. The initial five cycles of 50°C annealing can be eliminated if only primer sets with ≥ 50°C annealing temperature are used.

### Conventional PCR assays

The amplification of published SSRs and sequences harbouring SNPs by uniplex and multiplex PCR was performed under identical reaction conditions. PCR was performed in a 6 μl reaction mixture containing 0.2 mM dNTP, 1× *Tfi *PCR buffer (Invitrogen) (25 mM KCl, 75 mM (NH_4_)_2_SO_4_, 1 mM DTT, proprietary stabilizers, 50 mM Tris-HCl, pH 8.4), 1.5 mM MgCl_2_, 50 ng genomic DNA, 0.15 U Platinum *Tfi *DNA polymerase (Invitrogen), and 200 nmol each of standard length forward and reverse primer (see Additional File 1). The forward primers of published SSRs were labelled at their 5'-end with the fluorescent dye HEX (Applied Biosystems). For multiplex PCR, each primer set was added at a concentration of 200 nmol. Following an initial denaturation step of 2 min at 94°C to heat activate the DNA polymerase, PCR was performed for a total of 50 cycles with the touchdown profile: 30 s at 92°C, 60 s at (T_a_+10)°C, 60 s at 72°C, where T_a _was 50, 55 or 60°C, depending on the optimal conditions reported for the primer set. Following the first cycle, the annealing temperature was reduced by 0.5°C for the next 20 cycles. The PCR was finished with a final extension step of 10 min at 72°C.

### SSR analysis

Electrophoresis and visualization of SSRs was performed on a GelScan2000 (Corbett Research) and ABI3730 DNA analyser (Applied Biosystems). For analysis on the GelScan2000, PCR products were mixed with an equal volume of gel loading buffer (98% formamide, 10 mM EDTA and 0.5% basic fuchsin as tracking dye), heated for 3 min at 95°C, chilled quickly on ice and separated on a 4% sequencing gel [37]. For ABI3730 analysis, a standardized multi-pooling procedure was used to prepare SSR products amplified by multiplex PCR for electrophoresis. PCR products were diluted with five volumes of sterile water, pooled together at a ratio of 2:2:1:2 for VIC:FAM:NED:PET to give a final 20 μl volume, and desalted by ultra-filtration using an AcroPrep 384 filter plate with 10 kD Omega membrane according to the manufacturer's instructions (PALL Life Sciences). The desalted SSR products were resuspended in 25 μl of sterile water. Three μl of the resuspended SSR product was added to 8 μl of deionised formamide containing 0.8 μl of GelScan500 LIZ size standard (Applied Biosystems). The mixture was heated uncovered at 80°C for 5 minutes to evaporate the water and electrophoresed according to the manufacturer's instructions. This multi-pooling procedure resulted in 0.06 μl of each PCR product being electrophoresed. SSR allele sizing was performed using GeneMapper v3.7 software (Applied Biosystems). The pooling of PCR products with different dye-labels at the 2:2:1:2 ratio was to account for differences in the relative fluorescence of each fluorophore.

### SNP analysis

SNP assays were performed using allele-specific primer extension chemistry and xMAP™ technology (Luminex Corporation) on the BioPlex microsphere-based suspension array platform (BioRad). The assay was performed essentially as described by [38] and involved: (a) an initial PCR amplification of the target sequences harbouring the SNPs from genomic template, (b) purification of the PCR products to remove residual primer and dNTP, (c) assay of the SNPs using allele-specific primers with oligonucleotide capture probes at their 5'-ends, and (d) detection of the allele-specific primer extension products on the BioPlex instrument.

The multiplexed PCR products were diluted to 50 μl with sterile water and purified by ultra-filtration to remove excess PCR primer and dNTP using a Montage 384 PCR cleanup plate (Millipore) according to the manufacturer's instructions. The purified PCR products were resuspended in 20 μl of sterile water. Five μl of purified PCR product was added to a PCR well and dried by heating at 80°C for 15 min before 5 μl of allele-specific primer extension mix was added, which contained 1.25 μM each of dATP, dTTP, dGTP and biotin-14-dCTP (Invitrogen), 1.25 mM MgCl_2_, 1 × *Tsp *PCR buffer, 0.1 U Platinum *Tsp *DNA polymerase (Invitrogen), and 25 nmol of each allele-specific primer (see Additional File 1). The thermocycling conditions involved an initial denaturation step of 2 min at 94°C to activate the DNA polymerase, followed by 30 cycles of 30s at 94°C, 60s at 55°C, and 2 min at 74°C.

Following PCR, the allele-specific primer extension products were hybridised to fluorescently colour-coded carboxylated polystyrene LabMAP™ microspheres (Luminex Corporation). Each allele-specific primer contained an oligonucleotide sequence (ZipCode) at its 5'-end, allowing hybridization to a complementary sequence (called a cZipCode) covalently attached to the microsphere surface. The hybridization of the biotinylated allele-specific primer extension products for each SNP to their corresponding microspheres was carried out essentially as described by [39] in a 30 μl reaction volume containing 1× hybridization buffer (200 mM NaCl, 0.08% Triton X-100, 100 mM Tris-HCl, pH8.0) and 500 microspheres of each type. After initial denaturation at 95°C for 5 min and incubation at 37°C for 30 min, the microspheres were washed once with 100 μl of 1× hybridization buffer, resuspended in 50 μl of 1× hybridization buffer and conjugated with streptavidin by adding 250 μg of streptavidin-conjugated R-phycoerythrin (Invitrogen). The microspheres were incubated at room temperature for 15 min, washed once with 100 μl of 1× hybridization buffer, resuspended in 50 μl of 1× hybridization buffer and assayed on the BioPlex microsphere suspension array platform. The BioPlex instrument first identifies the specific microsphere (and thus the SNP locus) based on the microsphere colour then the presence or absence of the streptavidin-biotin conjugate (indicative of the presence or absence of the specific allele). The fluorescence on the surface of the microspheres resulting from the streptavidin label was converted to a median fluorescence intensity (MFI) value based on a minimum of 32 microspheres for each of the SNP alleles assayed. The allele present at each SNP locus was determined using BioPlex SNP Manager software v1.0 (BioRad).

## Authors' contributions

MJH developed the multiplex-ready PCR technology and experiments, and wrote the manuscript. TMN and AW carried out the experimental work, participated in the design of the study, and analysis and interpretation of data. All authors read and approved the manuscript

## Supplementary Material

Additional file 1 **Primer Information**. A Microsoft Excel workbook containing detailed information for primer sets used in the present study to amplify published SSRs and sequence-tagged-sites harbouring SNPs. This information includes published primer names and sequences, optimal locus-specific primer concentrations for multiplex-ready PCR, allele-size ranges and where each primer set was published in the literature. For the barley SNPs, the allele-specific primer capture probe sequences are also provided. Click here for file
